# Acupuncture for Japanese Katakori (Chronic Neck Pain): A Randomized Placebo-Controlled Double-Blind Study

**DOI:** 10.3390/medicina59122141

**Published:** 2023-12-09

**Authors:** Nobuari Takakura, Miho Takayama, Akiko Kawase, Ted J. Kaptchuk, Jian Kong, Mark Vangel, Hiroyoshi Yajima

**Affiliations:** 1 Department of Acupuncture and Moxibustion, Tokyo Ariake University of Medical and Health Sciences, 2-9-1 Ariake, Koto-ku, Tokyo 135-0063, Japan; takayama@tau.ac.jp (M.T.); yajima@tau.ac.jp (H.Y.); 2 Japan School of Acupuncture, Moxibustion, and Physiotherapy, 20-1 Sakuragaokacho, Shibuya-ku, Tokyo 150-0021, Japan; kawase@hanada.ac.jp; 3 Program in Placebo Studies & Therapeutic Encounter, Beth Israel Deaconess Medical Center, Harvard Medical School, 330 Brookline Avenue, Boston, MA 02215, USA; ted_kaptchuk@hms.harvard.edu; 4 Department of Psychiatry, Massachusetts General Hospital, Harvard Medical School, Charlestown, MA 02129, USA; kongj@nmr.mgh.harvard.edu; 5 Department of Radiology, Massachusetts General Hospital, Harvard Medical School, Charlestown, MA 02129, USA; mvangel@mgh.harvard.edu

**Keywords:** acupuncture, placebo, neck stiffness, shoulder stiffness, double blinding, randomized placebo-controlled trial, chronic neck pain

## Abstract

*Background and Objectives:* Although acupuncture is listed as a beneficial treatment for neck/shoulder stiffness, which has increased with the spread of information technology, to date, evidence of its efficacy under double-blind conditions has not been shown. This study aimed to assess whether acupuncture treatment with superficial skin piercing is superior to placebo treatment. *Materials and Methods:* A randomized, double-blind (practitioner–patient) placebo-controlled trial was performed at a single center with four arms (ISRCTN76896018). Four hundred patients with essential neck/shoulder stiffness were randomly assigned to penetrating needle treatment (acupuncture ritual and skin penetration), skin-touch needle treatment (acupuncture ritual and skin touch), no-touch needle treatment (acupuncture ritual alone), and no-treatment control. Each of the six acupuncturists applied a needle to each of the four acupoints in the neck/shoulder of 50 patients. *Results:* Each of the three treatments significantly (*p* = 0.01) improved neck/shoulder stiffness compared with the no-treatment control immediately and 24 h after treatment. There was a significant improvement in penetrating needle treatment over no-touch needle treatment 24 h later. However, there was no significant difference between the penetrating and skin-touch and skin-touch vs. no-touch. *Conclusions:* All treatments that received the ritual of acupuncture were better than the no-treatment control. Only genuine acupuncture involves the specific effects of needle insertion into the body. The acupuncture ritual had a significant impact on the subjective improvement of neck/shoulder stiffness; however, improvement with ritual alone versions of placebo acupuncture was not maintained as with superficial skin piercing. Our study provides important evidence of acupuncture efficacy and information regarding inert no-touch placebo control in acupuncture research.

## 1. Introduction

Acupuncture has been used to treat stiffness of the neck and shoulders (chronic neck pain), which is described as a feeling of discomfort such as heaviness, dullness, tightness, and rigidity of the muscles supporting the neck [1,2,3]. Neck/shoulder stiffness is classified as essential stiffness without any specific diseases or secondary stiffness associated with specific diseases [1,2]. This complaint is the second most frequent subjective symptom, followed by lumbago; for women and men, it is in first and second place, respectively, in Japan [4]. Although the prevalence of neck/shoulder stiffness is high, the current medical measures for neck/shoulder stiffness are insufficient [5,6].

Modern society is full of stresses and frustrations which cause an excessive increase in the muscle tone to develop stiffness in the neck and the shoulder muscles [7,8,9]. In particular, working with visual display terminals, personal computers, and tablets causes problems for industrial hygiene [7,10,11]. Considering that neck/shoulder stiffness has a strong impact on lowering work efficiency and quality of life, effective treatments for neck and shoulder stiffness have become increasingly important [7,10,11].

Acupuncture has been increasingly endorsed for the treatment of neck/shoulder stiffness, but further evidence is needed [12,13]. Needle insertion reduces stiffness with dull pain in the muscles in actual clinical settings. Previous placebo-controlled randomized controlled trials (RCTs) have also reported that acupuncture was more effective for chronic neck pain compared with placebo control [14,15], but many recommended researchers have called for a double-blind methodology to bolster the rigor of evidence. Furthermore, the effect of acupuncture in the treatment of non-specific chronic pain, including chronic neck pain, has been reported to be long-lasting [16,17]. Although the mechanisms underlying the effectiveness of acupuncture for neck/shoulder stiffness are not well understood, they may be explained by the following biological reactions caused by needle insertion: increase in local blood flow in the stiff muscle by axon reflex or somato-autonomic reflex, local analgesia by chemical mediators leaked from the damaged cells, activation of descending pain inhibitory pathways, or an inhibitory effect on motor neurons to innervate the stiff muscles [18].

As mentioned above, one of the shortcomings of acupuncture evidence is the absence of experiments conducted under double-blind (patient–practitioner blinding) conditions [10,11,19,20,21,22]. Many studies have considered that the current evidence of the efficacy of acupuncture on chronic pain, including neck/shoulder stiffness, is inconclusive because of the low quality of the methodology [23]. Previous RCTs using single-blind placebo/sham needles [24,25] have been criticized as insufficiently rigorous, and the evidence obtained under such blinding exclusively for patients is inconclusive [26,27,28,29,30,31,32,33]. In this study, we used double-blind needles to blind practitioners and patients, which have been validated for use in clinical settings [34,35], to overcome the limitations of single-blind RCTs [28]. Furthermore, we employed no-touch placebo needles without skin touching to compensate for the weakness of physiologically active skin-touch needles to avoid overlooking the specific effect of acupuncture compared with an inert placebo [35].

In this study, we enrolled patients who had essential neck/shoulder stiffness to emphasize the cause-effect relationship between needle insertion and improvement simply, clearly, and rigorously. This study aimed to assess whether acupuncture treatment with superficial skin piercing or skin touching had a specific effect over ritual alone for essential neck/shoulder stiffness under double-blind conditions.

## 2. Methods

The study protocol has been published previously [36]. This was a randomized, double-blind (practitioner-patient blind), placebo-controlled study performed in a single center with four arms: skin-piercing treatment with penetrating needles (penetrating treatment), skin-touching treatment with skin-touch placebo needles (skin-touch treatment), no skin-touch treatment with no-touch placebo needles (no-touch treatment), and control with no treatment (no-treatment control) [35].

The study protocol followed the CONSORT recommendations. The study was approved by the Ethics Committee of Tokyo Ariake University of Medical and Health Sciences (approval no. 11, date of approval: 8 July 2010). The trial was registered with ISRCTN76896018.

### 2.1. Setting and Participants

The study was conducted at Japan School of Acupuncture, Moxibustion, and Physiotherapy, and Tokyo Ariake University of Medical and Health Sciences, Tokyo, Japan.

We employed six (three males, three females) experienced and licensed acupuncturists (experience of acupuncture practice, mean ± standard deviation year: 12.5 ± 11.8 years), and recruit 400 volunteers with essential neck/shoulder stiffness. The study aim and protocol were explained to each practitioner and patient using a written consent form and the participants provided written consent.

#### 2.1.1. Inclusion of Volunteers

Patients who met the following criteria were included in the study:

1. Patients were 18 to 60 years of age.
2. Patients had functional neck/shoulder stiffness without pain due to specific diseases [1,2].
3. Patients had received acupuncture and had experienced de-qi.

#### 2.1.2. Exclusion of Volunteers

Patients who met the following criteria were excluded.

1. Patients with a plan within 24 h to receive acupuncture, massage, medication or any other treatment for neck/shoulder stiffness.
2. Patients with a plan within 24 h to do self-care for neck/shoulder stiffness, e.g., exercise, stretching and/or supplements.
3. Patients who had been diagnosed with any of the following diseases: cervical spondylosis, cervical hernia of the intervertebral disk, cervicobrachial disorder, thoracic outlet syndrome, hepato-cholecystopathy, high blood pressure, cerebrovascular disease, or cardiac disease [37,38].
4. Patients who had any neurological symptoms, such as paralysis or numbness in the neck, shoulder or upper extremities.
5. Patients who were diagnosed with diseases that produce neck/shoulder stiffness according to the last annual medical examination.
6. Systolic blood pressure was over 140 mmHg and/or diastolic pressure was over 90 mmHg just before the treatment.

### 2.2. Acupuncture Needles

**Figure 1:**
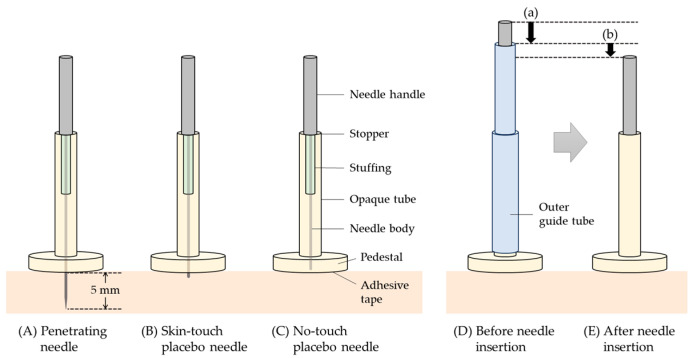
Three types of acupuncture needles for double blinding. (**A**) Penetrating needle for 5 mm insertion. (**B**) Skin-touch placebo needle. (**C**) No-touch placebo needle. (**D**) Before needle insertion. (**E**) After needle insertion. The bottom of the needle handle works as a stopper, so that the needle tip can reach a fixed depth. (a) Depth of skin penetration using tapping-in technique. (b) Depth of further insertion using alternating twirling technique after removal of outer guide tube [34,35,36].

We used three types of stainless needles for double blinding (Confidence Co., Ltd., Tokyo, Japan): (1) penetrating needles that can pierce the skin to a 5 mm depth; (2) skin-touch placebo needles, the tip of which can press against the skin but cannot penetrate it; and (3) no-touch placebo needles, the tip of which cannot reach the skin [34,35,36] (Figure 1). The diameter of the needles was 0.18 mm.

### 2.3. Randomization

**Figure 2:**
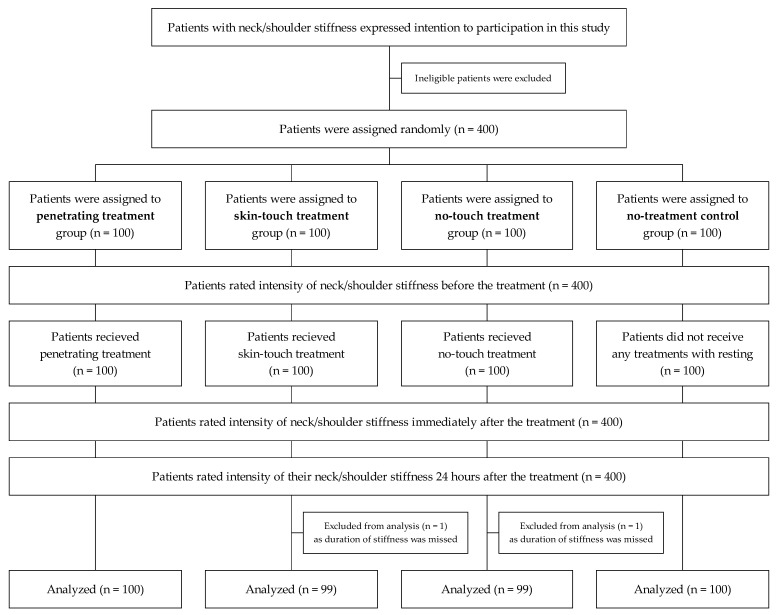
Trial flow diagram.

One of the authors randomly assigned 400 patients to the penetrating, skin-touch, no-touch, or no-treatment arms using a table of random numbers generated by the RAND function (Microsoft Office Excel 2011) (Figure 2). Each of the six acupuncturists treated 50 patients consecutively and in order.

### 2.4. Explanation to Patients

At the beginning of the study, we informed patients that they were going to be randomized to one of four groups: penetrating treatment, skin-touch placebo treatment, no-touch placebo treatment, or no-treatment control. If they were assigned to the no-treatment control group, they could receive real acupuncture treatment for neck/shoulder stiffness after completion of the trial if they desired.

### 2.5. Patients’ Evaluation of Neck/Shoulder Stiffness before Treatment

The patients recorded where the strongest stiffness was on the figure in which the neck and shoulder were drawn, and their duration (less than 2 weeks, 2 weeks to 3 months, and more than 3 months). The patients rated the intensity of neck/shoulder stiffness on a 100 mm visual analogue scale (VAS) ranging from 0 (no neck/shoulder stiffness) to 100 (the most severe neck/shoulder stiffness imaginable) [20,22].

### 2.6. Needle Application and Acupoints

For each treatment, the acupuncturist administered one needle to BL10, GB21, SI14, and BL42, in that order, on the side with the higher stiffness of patients sitting on a massage chair, using the tapping-in and alternating twirling techniques (Figure 1).

Ten minutes later, the acupuncturist removed the needle from the skin and placed it into an opaque envelope. If bleeding occurred, practitioners reported the incidence of bleeding. The needles were removed in the same order as they were applied.

### 2.7. Patients’ Evaluation after Treatment

The patients rated the intensity of stiffness immediately and 24 h after treatment on the VAS. In the no-treatment control group, the patients reported the same items [36].

The patients guessed whether the treatment was “penetrating,” “skin-touch” or “no-touch” or to record “cannot identify the treatment” and they reported their confidence level in their guesses on a 100 mm VAS ranging from 0 (no confidence) to 100 (full assurance) [34,35,36].

### 2.8. Practitioners’ Guess as to Treatment

After completion of each treatment, the acupuncturists guessed whether the treatment was “penetrating”, “skin-touch” or “no-touch” or to record “cannot identify the treatment”; they reported their confidence in their guesses on a 100 mm VAS ranging from 0 (no confidence) to 100 (full assurance) [34,35,36].

### 2.9. Outcomes

#### 2.9.1. Primary Measurements

Improvement in the neck/shoulder stiffness score immediately and 24 h after treatment were the primary outcomes. The decrease in stiffness VAS score of 10 mm or more was defined as the improvement based on the minimum clinically significant difference [39].

#### 2.9.2. Secondary Outcome Measurements

Practitioners’ and patients’ guesses at treatment and their confidence in their guesses were secondary outcomes.

### 2.10. Adverse Events

The patients, assistants, and acupuncturists reported adverse events with acupuncture treatments.

### 2.11. Statistical Analysis

With 100 patients in each group, we had 0.99 power to detect a significant difference within each of the four groups immediately and 24 h after treatment. For between-group comparisons, we had at least 0.98 power to detect a 10 mm minimum clinically significant difference in stiffness VAS score [39] between no-treatment control and each of penetrating, skin-touch, and no-touch treatments immediately and 24 h after treatment, based on a post hoc power analysis.

Baseline characteristics were analyzed using Fisher’s exact test (gender and duration of stiffness); one-way ANOVA (mean stiffness and mean age); and Levene’s test (SD stiffness and SD age).

A mixed-model regression was fit with stiffness a response and patient as a random effect. The fixed effects in this model were age, gender, duration, stiffness, group, and time, and all two- and three-factor interactions involving the last three of these covariates. Post hoc pairwise comparisons of group and times were performed to compare the effects of different interventions/conditions. The chi-squared test was used to compare numbers of improved patients among the four groups.

To evaluate the blinding effect, the mixed model was fit to data from each group separately (except no-treatment control), with stiffness as the response, participant as random effect, and covariates age, gender, stiffness duration, patient (or practitioner) guess, and time, as well as the two- and three-factor interactions of stiffness duration, patient guess, and time. The chi-squared test of goodness-of-fit was used to determine whether the proportions of penetrating, skin-touch, and no-touch in the patients guessed at each treatment were the same. The Kruskal–Wallis test was used to detect significant differences among the three treatment groups in the confidence levels of practitioners and patients guesses.

## 3. Results

### 3.1. Baseline Characteristics

The baseline values did not differ significantly between the randomization groups. There was no significant difference in stiffness before treatment between penetrating, skin-touch, and no-touch treatments (Table 1).

### 3.2. Improvement of Neck/Shoulder Stiffness Score Immediately and 24 h after the Treatment

Although all patients completed the study, the duration of stiffness was missing for two patients (both female, one randomized to no-touch treatment and one to skin-touch treatment); these patients were excluded from all analyses (Table 1). The effects of group (*p* < 0.01), time (*p* < 0.01), and their interaction (*p* < 0.01) were all significant. Improvements in neck/shoulder stiffness immediately and 24 h after were found in 69 and 74 patients with penetrating treatment, in 73 and 67 patients with skin-touch treatment, and in 61 and 59 patients with no-touch treatment, respectively. In the no-treatment control, 24 patients improved immediately, and 22 patients improved 24 h after treatment. The number of improved patients in the perpetrating treatment (χ^2^ = 54.17, *p* < 0.01), skin-touch treatment (χ^2^ = 49.26, *p* < 0.01), and no-touch treatment (χ^2^ = 28.77, *p* < 0.01) was significantly larger than that in the no-treatment control; however, no significant difference was observed among the three treatment groups (χ^2^ = 3.40, *p* = 0.18).

**Figure 3:**
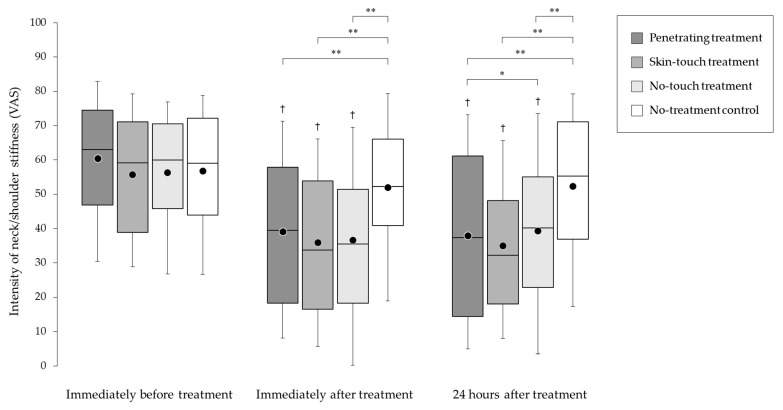
Changes in stiffness over time for the four treatments. The intensity of neck/shoulder stiffness was assessed using a 100 mm visual analogue scale (VAS). The top, middle, and bottom lines of the boxes correspond to the 75th, 50th, and 25th percentiles, respectively. The whiskers extend from 10th to the 90th percentile. The circles indicate the mean scores. * *p* < 0.05 and ** *p* < 0.01 indicate significant differences between groups. † *p* < 0.05 indicates a significant decrease in stiffness compared with immediately before treatment in each group.

The changes in stiffness over time for all treatments are shown in Figure 3. The penetrating, skin-touch, and no-touch treatments significantly (*p* < 0.01) improved neck/shoulder stiffness compared with the no-treatment control immediately and 24 h after treatment. There were no significant differences in improvement for penetrating vs. skin-touch placebo (*p* = 0.78), penetrating vs. no-touch placebo (*p* = 0.38), and skin-touch vs. no-touch placebo (*p* = 0.99) immediately after treatment. After 24 h, there was a significantly larger (*p* = 0.01) improvement in the penetrating treatment over the no-touch treatment, although no significant difference in improvement for penetrating vs. skin-touch (*p* = 0.75) and skin-touch vs. no-touch (*p* = 0.36) were observed. For all patients not randomized to no-treatment (*n* = 298), the change in stiffness with time was also significant (−35% post-treatment for all groups; −30% next day for no-touch, −37% next day for skin-touch and penetrating).

### 3.3. Effect of Blinding

For all 298 patients randomized to a group other than the no-treatment group, there was no significant association of practitioner guess with the randomization group (*p* = 0.33). On the other hand, there was a significant association of patient’s guess with group for these 298 patients (*p* = 0.001) (Table 2). Of the 298 treatments, 39 no-touch treatments and 2 skin-touch treatments did not elicit any needle sensations.

However, in 197 patients who were randomized to either skin-touch or penetrating acupuncture, and who did not report feeling no sensation at all, there was neither a significant association between practitioner guess and the randomization group (*p* = 0.80), nor was there a significant association between patient guess and the randomization group (*p* = 0.56).

**Figure 4:**
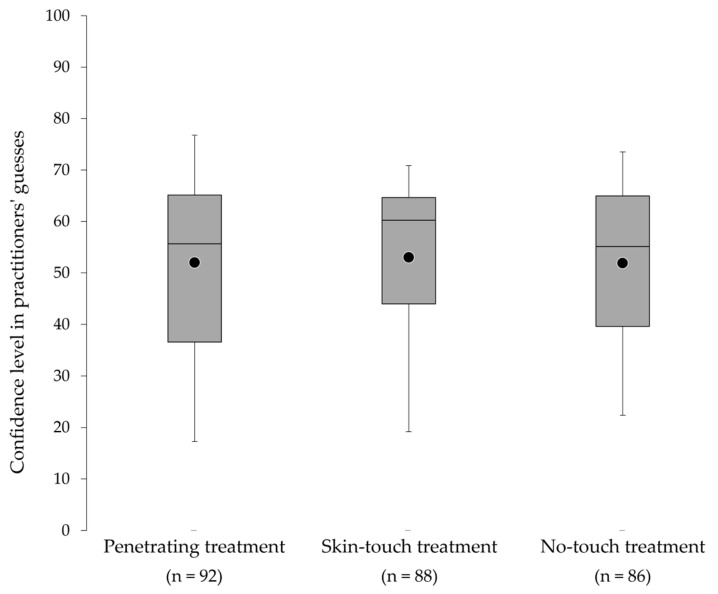
Confidence in practitioners’ guesses for each type of needle (except ‘cannot identify’). The top, middle, and bottom lines of the boxes correspond to the 75th, 50th, and 25th percentiles, respectively. The whiskers extend from the 10th to the 90th percentile. The circles indicate the mean scores.

**Figure 5:**
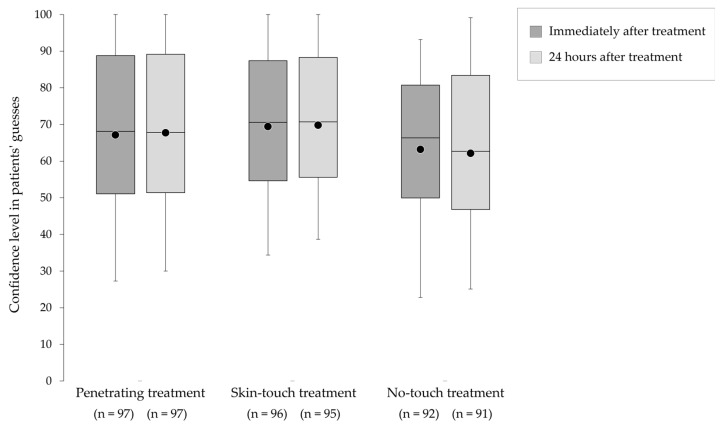
Confidence in patients’ guesses for each type of needle (except ‘cannot identify’). The top, middle, and bottom lines of the boxes correspond to the 75th, 50th, and 25th percentiles, respectively. The whiskers extend from 10th to the 90th percentile. The circles indicate the mean scores.

The median (mean ± standard deviation) scores of patients and practitioners confidence in their correct guesses were 66.9 (63.7 ± 27.3) and 54.3 (46.7 ± 24.9), respectively. Three patients correctly identified their treatment with certainty. There was no significant difference in the practitioners’ (*p* = 0.78) (Figure 4) and patients’ (*p* = 0.28) confidence excluding the ‘cannot identify the treatment’ between three treatments immediately after and 24 h after the treatment (Figure 5).

### 3.4. Adverse Events

The only adverse events were two very minor bleeding events.

## 4. Discussion

This is the first randomized placebo-controlled study to evaluate the efficacy of superficial needle insertion on essential neck/shoulder stiffness compared with skin-touch and no-touch placebo needles under double-blind conditions. No-touch treatment significantly improved the neck/shoulder stiffness as penetrating and skin-touch treatments. However, the efficacy of the no-touch treatment was not comparable to penetrating treatment one day later. The results indicated that the acupuncture ritual had a significant impact on the subjective improvement in neck/shoulder stiffness, and skin piercing had some effect on the acupuncture ritual.

In this study, we employed two kinds of placebo needles. To reveal the specific effect of skin piercing that is a characteristic component of acupuncture, a skin-touch placebo needle is indispensable. The blinding effect of skin-touch needles, which makes patients feel that they are receiving acupuncture, has been well validated [24,25,34,35,36]. In fact, to distinguish between penetrating acupuncture and skin touch, the analysis of the data excluded patients who reported feeling no sensation at all (*n* = 41); only two of these patients were randomized to skin-touch and none to penetrating acupuncture in the present study, supporting the hypothesis that the needle is effectively double-blind. The rationale for excluding patients who did not report feeling of any sensations in the above analysis was that these patients were told that there was a “no-touch” randomization group, and most of them correctly guessed that they were indeed randomized to that group (39/41 = 95%). This is an artifact of the study design, and does not appear to be related to the performance of the experimental needle. The discrepancy in the statistical analysis for patient blinding between 197 patients excluded patients who felt no acupuncture sensation and 298 patients receiving all-inclusive treatment was due to the 39 receiving no-touch treatments.

However, it should be noted that skin pressing with a nonpenetrating acupuncture needle is physiologically active; thus, a skin-touch placebo is argued to be an inappropriate control to reveal the efficacy of acupuncture [40,41]. The efficacy of the skin-touch needle has the potential to lead us to overlook acupuncture efficacy in cases where skin pressing with non-penetrating needles is defined as a noninvasive type of acupuncture. Therefore, despite the difficulty in patient blinding, we included a no-touch placebo needle arm as an inert placebo control to determine the efficacy of the ritual alone to compensate for the limitations of the skin-touch placebo needles.

As for the efficacy with no-touch treatment, at which there were only a few patients’ correct guesses with certainty, as well as the other two treatments, the same level of significant improvement was comparable to the other two treatments immediately after. The efficacy of the no-touch treatment could be attributed to the equivalent amount of placebo effect of the acupuncture ritual in the penetrating and skin-touch treatments. The comparable improvement with no-touch treatment to the other two treatments despite a larger number of correct patients’ guesses on no-touch treatments than the other treatments indicates that usage of placebo device per se was critical to reduce bias, but not for whether the patient’s guess was correct or not. This speculation is supported by the meaningful efficacy of reducing subjective complaints observed in an open level placebo study in which the participants were clearly explained that they would take an inactive (i.e., ‘‘inert’’) placebo such as a sugar pill prior to treatment [42]. The results of this previous study suggest that the use of a placebo control per se is important for the development of a placebo effect and that the success or failure of blinding patients to the intervention is irrelevant. If the open-label placebo acupuncture would induce clinically meaningful placebo effects and consensus would be given to no-touch treatment as placebo acupuncture, it can be said that the tissue damage with skin piercing had a specific component to maintain improvement of neck/shoulder stiffness from the results one day after.

However, it was also demonstrated that superficial skin piercing of 5 mm depth did not have a specific effect over skin press with a skin-touch needle. The reason for this might be that input to the central nervous system with a skin-touch needle was very similar to 5 mm needle insertion; in fact, acupuncture sensation including skin penetration pain with a 5 mm needle insertion and skin-touch needle are comparable [34]. Tissue damage with skin piercing, which is a prominent difference between penetrating and skin-touch needles, might contribute to the maintenance of its efficacy. To scrutinize this point, a comparison should be carried out between efficacy with more than 5 mm insertion and skin-pressing, skin-touching, and no-touch needles to reveal the specific effects of skin piercing, skin pressing, and skin touching, which also leads to the exploration of the most appropriate placebo needle. Precise differentiation of these components is possible by adjusting the length of the blunt tips protruding from the bottom of the guide tube using the double-blind needles [34,35].

From the current results, the patients in the three groups had the same level of expectation, which plays a key role in placebo effects, arising from the ritual of acupuncture; moreover, placebo effect might be largely involved in the efficacy of each treatment [43]. Placebo acupuncture with expectation induced a more powerful effect than overt placebo acupuncture on the brain activities of the dorsolateral prefrontal cortex, anterior cingulate cortex, and midbrain which activates the endogenous pain modulatory descending pathway [43]. The placebo mechanisms of sensory modulation, as seen in placebo analgesia, might mainly contribute to the improvement in neck/shoulder stiffness with the three treatments. Furthermore, motor function, including muscle rigidity, was improved with placebo treatment in patients with Parkinson’s disease [43]. The involvement of such a mechanism that modulates motor function with placebo treatment cannot be ruled out. Considering that acupuncture may have both specific and placebo effects [43], the possible involvement of acupuncture-specific effects cannot be disregarded. The following are possible mechanisms of acupuncture to improve stiffness of the muscles: local analgesia by chemical mediators leaked from the damaged cells, activation of descending pain inhibitory pathways, or an inhibitory effect on motor neurons to innervate the stiff muscles [18], and an increase in local blood flow in the stiff muscle by axon reflex or via somato-autonomic reflex. However, improvement in local blood flow seems unlikely, considering previous results in which dry needling into the trapezius did not increase blood flow in this muscle in neck/shoulder patients [20]. Further research is needed to determine the mechanism of improvement in stiffness of the neck and shoulder with placebo or acupuncture.

Given that neck/shoulder stiffness is increasingly prevalent in a highly stressful society, perhaps because of computer use [44,45,46], the following may be sufficient reasons to recommend acupuncture as a salubrious treatment for essential neck/shoulder stiffness to interested patients [47,48,49,50]: negative socioeconomic consequences of neck/shoulder stiffness, such as functional incapacity to sick leave [7], no established cure [5,6], acupuncture with few adverse events [51], more than 30% acupuncture ritual-specific improvement in no-touch treatment [39], and significant efficacy with penetrating treatment over no-touch treatment one day later.

The limitations of this study include only a single treatment session, short follow-up, and shallow insertion; the patients knew that the two types of placebo needles were options. Further research is warranted with multiple treatments using more needles and/or deeper insertion while telling the patient that there is one placebo arm and not two.

## 5. Conclusions

In this four-arm study, we used genuine acupuncture to penetrate the skin, placebo acupuncture to touch the skin, placebo acupuncture not touching the skin, and a no-treatment control. All three treatment arms received ritual of acupuncture. Only genuine acupuncture involved the specific effects of needle insertion into the body.

All treatments were better than those in the no-treatment control. The ritual of acupuncture had a significant impact on the subjective improvement of neck/shoulder stiffness, but improvement with ritual-alone versions of placebo acupuncture was not maintained as with the superficial skin piercing. Our study provides important evidence on acupuncture efficacy (it is more effective than no treatment) and important information on placebo controls in acupuncture research (the efficacy of physiologically inert no-touch treatment was larger than that of no-treatment).

## Figures and Tables

**Table 1:** Baseline characteristics.

	Arms of Acupuncture Treatments				*p*-Value *
	Penetrating Treatments	Skin-Touch Treatments	No-Touch Treatments	No-Treatment Control	
Patients included in analysis (number)	100	99	99	100	
Age (mean (SD) in years)	29.5 (8.6)	29.1 (8.3)	29.2 (10.2)	28.3 (8.2)	0.81 (0.44)
Gender (numbers: male/female)	41/59	41/58	44/55	33/67	0.4
Duration of neck/shoulder stiffness before treatment (numbers: ≤2 weeks/<2 weeks and <3 months/≥3 months)	23/15/62	29/17/53	18/24/57	30/20/50	0.27
Intensity of neck/shoulder stiffness before treatment (mean (SD) on 100 mm visual analogue scale)	60.7 (19.5)	56.0 (19.9)	56.5 (18.8)	57.0 (18.9)	0.3 (0.92)

* Note: *p*-values are from Fisher’s exact test (gender and duration of stiffness); one-way ANOVA (mean stiffness and mean age); and Levene’s test (SD stiffness and SD age).

**Table 2:** Number of participants’ guesses to treatment.

	Arms of Acupuncture Treatments			*p*-Value *
	Penetrating Treatments	Skin-Touch Treatments	No-Touch Treatments	
Patients’ guesses to treatment (‘penetrating’/‘skin-touch’/‘no-touch’/‘cannot identify’)				
Immediately after treatment (number)	83/14/0/3	75/19/2/3	26/27/39/7	<0.01
24 h after treatment (number)	80/14/3/3	74/18/3/4	28/25/38/8	<0.01
Practitioners’ guesses to treatment (‘penetrating’/‘skin-touch’/‘no-touch’/‘cannot identify’)	30/35/27/8	29/36/23/11	17/38/31/13	0.39

* *p*-values are obtained from Pearson’s chi-square test.

## Data Availability

The data presented in this study are available on request from the corresponding author. The data are not publicly available due to currently no available depository system in place in our university.
